# The CO_2_ Storage Capacity of the Intercalated Diaminoalkane Graphene Oxides: A Combination of Experimental and Simulation Studies

**DOI:** 10.1186/s11671-015-1026-9

**Published:** 2015-08-08

**Authors:** Jing Xu, Wei Xing, Lianming Zhao, Feifei Guo, Xiaozhong Wu, Wenbin Xu, Zifeng Yan

**Affiliations:** College of Science, China University of Petroleum, Qingdao, Shandong 266580 People’s Republic of China; State Key Laboratory of Heavy Oil Processing, Key Laboratory of Catalysis, China University of Petroleum, Qingdao, 266580 People’s Republic of China

**Keywords:** Intercalated graphite oxides, CO_2_ adsorption, Grand canonical Monte Carlo simulation

## Abstract

To study the effect of interlayer spacing of pillared graphene oxides (GOs) on CO_2_ uptake, we have obtained CO_2_ isotherms with respect to the interlayer distance of pillared graphene oxide by both experimental and simulation methods. Interlayer distances of GO were modulated by intercalation of three kinds of diaminoalkanes with a different number of carbon atoms (NH_2_(CH_2_)_*n*_NH_2_, *n* = 4, 8, and 12) as pillars. The intercalated GOs (IGOs) and their reduced products (RIGOs) are characterized using a variety of approaches such as X-ray powder diffraction (XRD), Fourier transform infrared spectroscopy (FT-IR), X-ray photoelectron spectroscopy (XPS), and N_2_ adsorption. Gas adsorption performance shows that the CO_2_ uptake of IGOs and RIGOs decrease with the increase of the interlayer distance at low pressure, while at high pressure, the adsorption capacity of IGO-12 has a larger growth than those of both IGO-4 and IGO-8 and surpasses them at 30 bar. The contribution of the electrostatics to CO_2_ adsorption is larger than that of van der Waals force at low pressures, whereas for the high pressures, the adsorption is dominated by van der Waals force.

**Electronic supplementary material:**

The online version of this article (doi:10.1186/s11671-015-1026-9) contains supplementary material, which is available to authorized users.

## Background

With the economic growth and industrial development, the excess fossil fuel combustion leads to a rapid increase of global warming and climate change. The rising atmospheric levels of CO_2_ are considered to be responsible for the warming effect on the climate, because CO_2_ emissions account for ca. 70 % of the gaseous irradiative force causing the greenhouse effect [1, 2]. Therefore, reducing the anthropogenic emission of CO_2_ has recently become a political and technological priority [3]. Efficient CO_2_ capture from existing emission sources plays a crucial role in reducing greenhouse gases in large quantities. However, CO_2_ has a very low density under ambient conditions and thus is very difficult to be stored. Among various methods, adsorptive storage and capture of CO_2_ by physical adsorption in porous media is considered as an energetically efficient and technically feasible approach, where the gas sorption and storage capacity is mainly governed by a large accessible surface area and pore structure [4]. A wide variety of tailor-made porous materials, such as various carbon-based adsorbents (e.g., activated carbons [5, 6], carbon nanotubes [7, 8], and graphene [9]), zeolites [10, 11], and metal−organic frameworks (MOFs) [12, 13] have been proposed and studied for CO_2_ storage application. Among them, graphene and graphene-based materials are considered as very promising candidates for the adsorption and storage of CO_2_ [14] due to their unique properties such as large theoretical specific surface area and structural and chemical tenability. For example, Lee et al. reported an adsorption of 6.4 mmol g^−1^ of CO_2_ at 30 bar and 298 K on exfoliated graphene oxide (GO) with a specific surface area (SSA) of 547 m^2^ g^−1^ and a total pore volume of 2.47 cm^3^ g^−1^ [15]. Mishra and Ramaprabhu found that a hydrogen-exfoliated graphene with a SSA of 443 m^2^ g^−1^ shows an enhanced CO_2_ adsorption of 21.6 mmol g^−1^ at 11 bar and 298 K [16]. Meng and Park developed a kind of vacuum exfoliated graphene nanoplates with a high capture capacity, up to 56 mmol g^−1^, at 30 bar and 298 K [17]. They also found that the improved CO_2_ capture capacity of the graphene nanoplates is attributed to the larger interlayer spacing and higher interior void volume [17].

However, most of the pure graphene materials prepared seem very difficult to reach the theoretical specific surface area (2600 m^2^ g^−1^ [14]) and realize expanded graphene layers without any supports inserted between them. Therefore, pillaring of graphene or graphene oxide with organic ligands has been considered [18]. Recently, Zhou et al. designed and fabricated a porous graphene material by linking non-planar terpyridine complexes through an azide–alkyne click reaction [19]. This complex possesses high specific surface area of 440 m^2^ g^−1^, and its carbon dioxide capacity could reach up to 2.6 mmol g^−1^ at 273 K and 1 atm. Burress et al. [20] developed a novel pillared graphene oxide framework (GOF) material by cross-linking the benzenediboronic acids between GO layers. The GOF material shows the maximum interlayer distance of 1.05 nm and SSA of 470 m^2^ g^−1^ and presents a good CO_2_ adsorption of ~2.7 mmol g^−1^ at 4 bar and room temperature [20]. All these demonstrate that interlayer spacing of graphene-based materials could be tuned using pillaring molecules and thus remarkably influences their gas adsorption capacity. However, to the best of our knowledge, the investigations are rather scarce for dealing with the evolution of CO_2_ adsorption properties with interlayer spacing of graphene-intercalated materials.

In this work, we investigated CO_2_ uptake for a wide range of interlayer distances using the three-dimensional structure of GO obtained from intercalation of diaminoalkanes (H_2_N(CH_2_)_*n*_NH_2_). The interlayer spacing of intercalated composites was controlled precisely by adjusting the number of methylene units in H_2_N(CH_2_)_*n*_NH_2_ (*n* = 4, 8, and 12). The effect of structural parameters of intercalated composites on their CO_2_ adsorption properties was studied by a combination of experiment and grand canonical Monte Carlo (GCMC) simulation.

## Methods

### Experimental Section

#### Material Preparation

Graphite powder (~1.5 μm) was purchased from Qingdao Ruisheng Graphite Co., Ltd. The diaminoalkanes were purchased from Aladdin Chemical Reagents Company. All others reagents were purchased from Shanghai Chemical Reagents Company and used as received. Graphite oxide was prepared according to a modified Hummers method [21]. GO suspension (6 mg ml^−1^) was prepared by dispersing graphite oxide in deionized water under ultrasonication for 1 h. The diaminoalkane-intercalated graphene oxides (IGOs) were synthesized according to Margarita’s method [22]. In a typical synthesis, 4.16 mmol of 1,*n*-diaminooctane (where *n* represents the number of methylene units in diaminoalkanes) was dissolved into 35 mL of ethanol under stirring. The resulting solution was added into 33 ml of the as-prepared GO suspension under vigorous stirring at ambient temperature. The reaction continued for 48 h at room temperature with continuous stirring. Afterwards, the resulting solution was isolated by centrifugation, washed sequentially with deionized water/ethanol mixture (1:1 volume ratio) four times, then filtered, and dried in a vacuum oven at 80 °C for 24 h. The as-synthesized IGOs were then reduced by hydrazine hydrate at room temperature and dried at 50 °C under vacuum. The resultant was designated as reduced IGO (RIGOs).

#### Material Characterization

The interlayer distances of IGOs and RIGOs were examined by X-ray powder diffraction (XRD; PANalytical B.V., Netherlands) using a Cu Kα_1_ radiation (0.15405 nm). The surface properties of the samples were characterized using Fourier transform infrared spectroscopy (FT-IR; Nicolet 6700, USA) and X-ray photoelectron spectroscopy (XPS; PHI 5000 VersaProbe, ULVAC-PHI, Japan). Nitrogen adsorption–desorption isotherms were measured at liquid nitrogen temperature (77 K) and CO_2_ adsorption was performed at 273 K and 298 K using a surface area and porosity analyzer (ASAP2020M, Micromeritics, USA). The carbon samples were degassed under turbomolecular vacuum before sorption measurements. N_2_ and CO_2_ gases with super high purity (99.999 %) were used for the physisorption measurements. The Brunauer, Emmett, and Teller (BET) equation was used to calculate the apparent surface area from N_2_ adsorption data obtained at *P*/*P*_0_ between 0.05 and 0.2. For advanced porosity analysis, pore size distributions and cumulative pore volumes were determined by using non-local density functional theory (NLDFT) method considering sorption of CO_2_ at 273 K in carbon as a model adsorbent and slit-like pores as a pore model. The implemented NLDFT model was supplied by the Quantachrome Autosorb ASiQwin 2.0 software. Note that microscopic methods based on statistical mechanics, such as NLDFT, which allow describing the configuration of the adsorbed phase on a molecular level, are currently considered as the more accurate method.

### Theoretical Section

#### The Model Structure of IGOs

The periodic models of [C_36_O_2_(OH)_7_(HN(CH_2_)_4_NH)]_4_, [C_32_O(OH)_5_(HN(CH_2_)_8_NH)]_4_, and [C_28_O(OH)_3_(HN(CH_2_)_12_NH)]_4_ are representative of IGO-*n* (*n* = 4, 8, and 12) structures, respectively (Additional file 1: Figure S1). The chemical composition of the models was similar to that indicated by the experimental measurement of diaminoalkane, epoxy, and hydroxyl. The models were generated by using the periodic density functional theory (DFT) calculation, which were performed using the PW91 GGA functional with the double numerical basis set containing polarization functions (DNP) available in the DMol^3^ code packed in the Materials Studio (MS) 5.0 package [23, 24].

#### Interatomic Potentials

In this study, CO_2_ molecule was represented by the conventional rigid linear triatomic model with three charged LJ interaction sites (C–O bond length of 0.1149 nm) centered on each atom as developed by Harris and Yung (see Additional file 1: Table S1) [25]. The interactions between the adsorbates and IGOs were described by a combination of site–site LJ and Coulombic potentials. In this work, the universal force fields (UFF) [26] (see Additional file 1: Table S1), which have been widely used to predict the thermodynamic and dynamic properties of various guests in graphene materials, were employed to model the atoms of IGOs. All the LJ cross-interaction parameters between the adsorbate/adsorbate and adsorbate/IGOs were determined by the Lorentz–Berthelot mixing rule, i.e*.*, *ε*_*ij*_ = (*ε*_*ii*_*ε*_*jj*_)^1/2^, *σ*_*ij*_ = (*σ*_*ii*_ + *σ*_*jj*_)/2.

#### Atomic Partial Charge for IGOs

The Mulliken charges were used to simulate the adsorption isotherms of CO_2_ in the IGOs. These charges were obtained from the periodic DFT calculation, which were performed on the optimized unit cells of IGO using the PW91 GGA functional and the DNP basis set with the DMol3 code packed in the MS 5.0 package [23, 24].

#### Simulation Details

Grand canonical Monte Carlo (GCMC) simulations were conducted to explore the adsorption behaviors of CO_2_ in the graphene using the MuSic code that was developed by the Snurr group from the Western University (USA) [27]. For the simulations of CO_2_, four types of attempts are considered: (i) insert, (ii) delete, (iii) transport, and (iv) rotate. The simulation box consisted of 8 (2 × 2 × 2) unit cells for the IGO-4, 24 (2 × 6 × 2) unit cells for the IGO-8, and 12 (3 × 2 × 2) unit cells for the IGO-12 materials. A cutoff radius of 1.2 nm was applied to the LJ interactions, while the long-range electrostatic interactions were handled by the Ewald summation method. Periodic boundary conditions (PBC) were considered in all the three dimensions. The Peng–Robinson equation of state was used to convert the pressure to the corresponding fugacity that was used in the GCMC simulations. For each state point, GCMC simulations consisted of 1 × 10^7^ steps to ensure the equilibration, followed by 1 × 10^7^ steps to sample the desired thermodynamic properties.

## Results and Discussion

### Physicochemical Properties

**Fig. 1:**
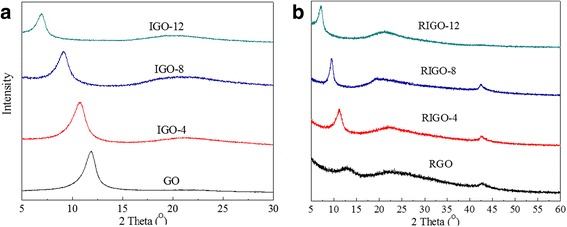
XRD patterns of **a** GO and IGOs and **b** RGO and RIGOs

The XRD patterns of GO, IGOs, and RIGOs are shown in Fig. 1. As shown in Fig. 1, GO exhibits a strong and sharp diffraction peak at 2*θ* = 11.9°, which corresponds to the diffraction of the (001) plane with an interplanar distance of 0.75 nm. The diffraction peak corresponding to the (002) plane of graphite at about 2*θ* = 26.5° is not observed, suggesting the complete oxidation of graphite. The oxidation of graphite leads to a large increase in the interplanar distance from about 0.34 to 0.75 nm. This is because oxygen-containing functional groups were attached to both sides of each graphene layer during oxidation. The reflection peaks of IGO-4, IGO-8, and IGO-12 appear at 10.8°, 9.1°, and 6.9°, respectively, exhibiting an increasing shift in the (001) peak position toward low angles. These suggest the one-dimensional expansion of the GO layers along its *c*-axis with an increased *d*-spacing as high as 0.82 (IGO-4), 0.97 (IGO-8), and 1.28 nm (IGO-12) (see Table 1). The significant increase of interlayer distances of IGOs indicates that the diaminoalkanes are inserted into lamellar GO sheets. With the increase of methylene units in diaminoalkanes from 4 to 12, IGOs exhibit a gradually increasing and well-defined *d*-spacing between GO layers, suggesting that interplanar space is sensitive to the length of diaminoalkanes. In addition, it should be noted that the interlayer spacing is smaller than the theoretical size of intercalated diaminoalkanes, because the alkyl chains of intercalated diaminoalkanes may be bent and/or inclined.

**Table 1:** Physicochemical properties of GO, RGO, IGOs, and RIGOs

Samples	XRD *d*-spacing (nm)	*S*_BET_^a^ (m^2^ g^−1^)	*S*_mic_^b^ (m^2^ g^−1^)	Cumulative pore volume of CO_2_ (cm^3^ g^−1^)
GO	0.75	–	–	–
RGO	0.69	–	–	–
IGO-4	0.82	4.7	194.1	0.036
RIGO-4	0.79	14.0	334.7	0.053
IGO-8	0.97	10.6	138.4	0.022
RIGO-8	0.94	12.1	148.1	0.017
IGO-12	1.28	14.1	132.5	0.009
RIGO-12	1.24	26.1	118.5	0.008

^a^BET surface area determined by N_2_ sorption (77 K)

^b^Cumulative micropore surface area determined by CO_2_ sorption (273 K) using the DFT method with a slit-shaped pore model

To further modulate the interlayer distance, the reduction by hydrazine hydrate was performed at room temperature. As shown in Fig. 1, RIGOs show two new reflection peaks at 23° (002) and 42.4° (100), suggesting partial reduction of GO. The main reflection peaks of the RIGO-4, RIGO-8, and RIGO-12 appear at 11.3°, 9.5°, and 7.2°, with corresponding interplanar distances of 0.79, 0.94, and 1.24 nm (Table 1), respectively. The interplanar distances of RIGOs are slightly shorter than that of IGOs due to the removal of oxygen-containing groups that weaken the steric effect. However, the pillared structures of RIGOs are maintained after hydrazine reduction at room temperature.

**Fig. 2:**
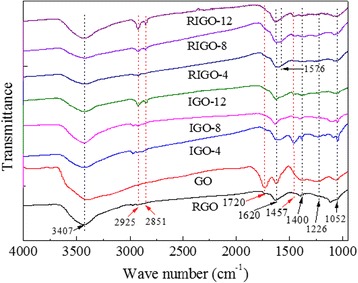
FT-IR spectra of GO, RGO, IGOs, and RIGOs

FT-IR analyses are adopted to investigate the evolution of functional group during the intercalation of GO (see Fig. 2). For GO, one can see the following vibrational bonds: the broad O−H stretching vibration at 3407 cm^−1^, the sharp C=O stretching vibration at 1720 cm^−1^, the peak at 1400 cm^−1^ due to the O−H bending vibrations from hydroxyl group, and the peaks at 1226 and 1052 cm^−1^ corresponding to C−O stretching vibrations of epoxy and alkoxy. Furthermore, there is an additional peak located at 1620 cm^−1^, which can be attributed to C=C stretching or skeletal vibrations of unoxidized graphitic domains. After intercalation of diaminoalkanes, IGOs show a clear, distinguishable additional IR mode at 1457, 2851, and 2925 cm^−1^, corresponding to bending vibration, symmetric, and asymmetric stretching of the methylene groups (−(CH_2_)_*n*_−), respectively. All these further indicate that the diaminoalkanes are intercalated into the layers of GO. In addition, compared to GO, the peak intensity of C–O and C=O groups of IGO decreases significantly, indicating that the GO surface functional groups were partially removed during the intercalation process. After reduction of IGOs, there is a new peak at 1576 cm^−1^ corresponding to the skeletal vibration of graphene sheets. This may be caused by the partial restoration of graphitic structure after hydrazine reduction.

**Fig. 3:**
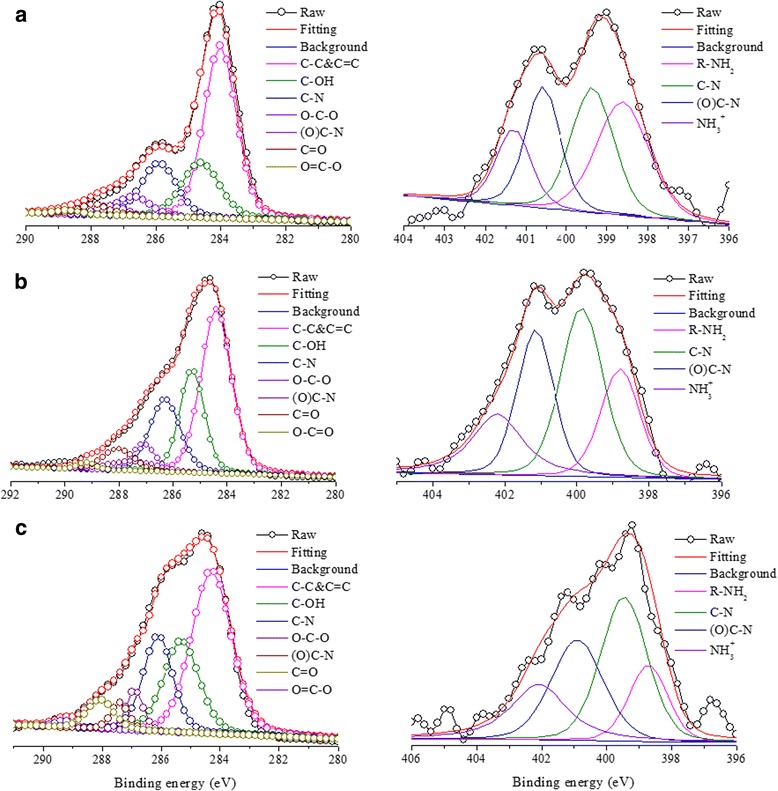
C 1s (*left*) and N 1s (*right*) XPS spectra of **a** IGO-4, **b** IGO-8, and **c** IGO-12

The surface chemical properties for GO and IGOs were also revealed by XPS study Fig. 3 and Additional file 1: Figure S2. The full-range XPS analysis of GO clearly shows the presence of carbon (C) and oxygen (O) with atomic percentages of 69.7:30.3. The C 1s spectrum of GO can be deconvolved into five peaks at 284.6, 285.0, 286.7, 287.6, and 289.0 eV (Additional file 1: Figures S2 and Table S3), corresponding to C=C/C–C in aromatic rings, C–O in alkoxyl groups, O–C–O in epoxyl groups, C=O in carbonyl groups, and O–C=O in carboxyl groups, respectively. After intercalation of diaminoalkanes, the peak intensity of O–C–O in IGOs decreased sharply, whereas that of the C–OH species increased (see Fig. 3). Concurrently, the two new peaks for C–N and N–C(O) bonds appear as shown in the C 1s spectra, corroborating with its N 1s XPS spectra and suggesting that a covalent bond-forming reaction has occurred. This behavior can be understood via the mechanism suggested by Burlinos [28], where alkylamine could readily react with graphene oxide via nucleophilic attack of amine on epoxide group. Interestingly, with the increase of the number of methylene in pillaring units, the content of C–OH species increases gradually, but that of C–O–C species decreases (Table 2). This can be attributed to the different lengths of diaminoalkanes (*n* = 4, 8, and 12) and their possible conformations between GO layers. With the increase of the length of alkyl chains, there is a larger probability of amines binding with epoxide groups on GO, forming formbridge or loop conformations. This situation is also supported by the N 1s XPS spectra, where the C–N and (O)C–N species increase and the R–NH_2_ groups decrease with the number of methylene increasing in diaminoalkanes (Table 3). A weak alkylammonium ion (C–NH_3_^+^) peak is also present in the N 1s spectra of IGOs, which originated from the association of amine with the small number of carboxylate groups on the edges of the graphene oxide sheets, as proposed by Matsuo et al*.* [29]. In addition, for IGO-*n* (*n* = 4, 8, and 12), the atomic ratio of C, N, and O is found to be 76.2:3.7:20.1, 78.7:4.0:17.3, and 81.6:3.6:14.9, respectively, suggesting that a ratio of graphene C, diaminoalkane pillar, and oxygen-containing group is about 37.0:1:10.8, 31.3:1:8.6, and 29.7:1:4.7, respectively.

**Table 2:** The C 1s XPS spectra of IGOs

Bonds	Peak area		
	IGO-4 (%)	IGO-8 (%)	IGO-12 (%)
C–C/C=C	50	44	45
C–OH	19	22	23
C–N	17	20	20
C–O–C	6	5	4
N–C(O)	2	4	3
C=O	3	5	5
O–C=O	1	1	1

**Table 3:** The N 1s XPS spectra of IGOs

Bonds	Peak area		
	IGO-4 (%)	IGO-8 (%)	IGO-12 (%)
R–NH_2_	32	20	15
C–N	29	34	35
(O)C–N	23	26	28
C–NH_3_^+^	14	18	20

**Fig. 4:**
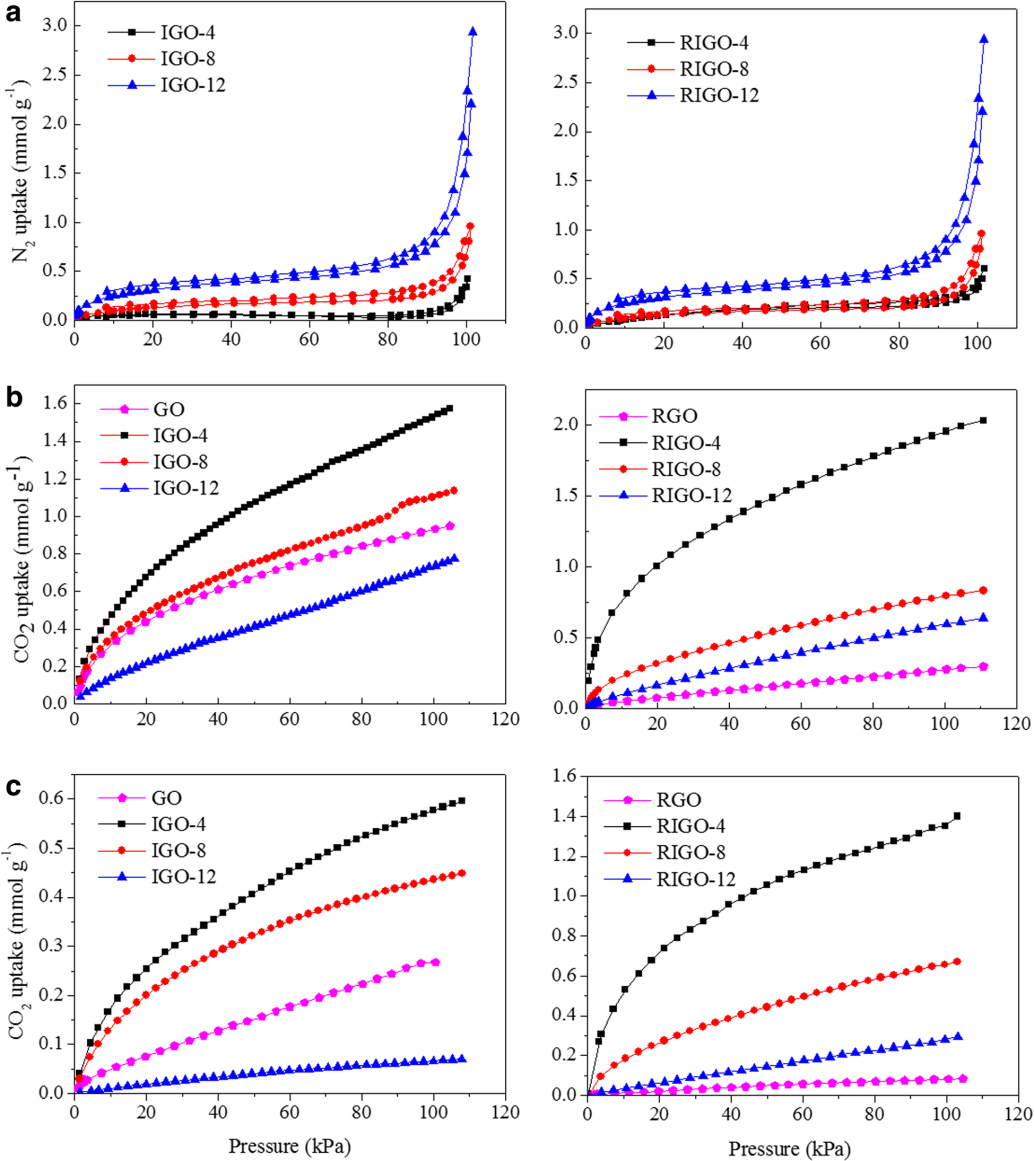
N_2_ adsorption–desorption isotherms at **a** 77 K and CO_2_ adsorption isotherms at **b** 273 and **c** 298 K for (*left*) GO and IGOs and (*right*) RGO and RIGOs

Nitrogen adsorption experiments (at 77 K) were performed to examine the surface textural characteristics. Fig. 4a shows the N_2_ adsorption–desorption isotherms, which fall in between I- and IV-type isotherms. At low relative pressure, the adsorption amount rapidly increases, suggesting the existence of micropores in IGOs. On the other hand, the H1 and H2 hysteresis loops and the durative increase of the adsorption capacity at *P* > 40 kPa for IGOs reveal the presence of meso/macropores formed by accumulation of IGO sheets. When the number of methylene units in diaminoalkanes rises from 4 to 8, and to 12, the amount of adsorbed N_2_ at 1 atm gradually increases from 0.42 to 1.30 and then to 1.54 mmol g^−1^, respectively. The corresponding BET surface areas increase from 4.7 m^2^ g^−1^ for IGO-4, to 10.6 m^2^g^−1^ for IGO-8, and to 14.1 m^2^g^−1^ for IGO-12 (Table 1). After hydrazine reduction, the adsorption capacity of N_2_ in the RIGOs increases slightly. The N_2_ uptake at 1 atm increases to 1.52 mmol g^−1^ for RIGO-4, to 2.92 mmol g^−1^ for RIGO-8, and to 9.00 mmol g^−1^ for RIGO-12, and the corresponding BET surface area is found to be 14.0, 12.1, and 26.1 m^2^ g^−1^, respectively.

### CO_2_ Capture Performances

**Fig. 5:**
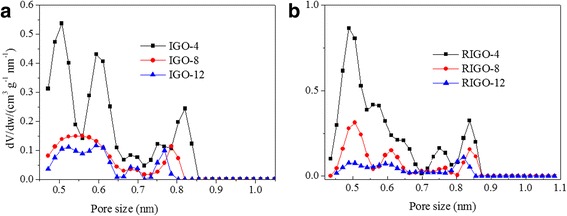
The pore size distribution curves of **a** IGOs and **b** RIGOs determined by CO_2_ sorption using the NLDFT method

The CO_2_ adsorption performances were measured at 273 and 298 K under the pressure of 0~1 atm (see Fig. 4). In contrary to the adsorption of N_2_, the total CO_2_ uptake follows the order of IGO-4 (~1.54 mmol g^−1^ at 1 atm) > IGO-8 (~1.11 mmol g^−1^) > GO (~0.93 mmol g^−1^) > IGO-12 (~0.74 mmol g^−1^) at 273 K (see Fig. 4b). Compared with GO, intercalation of a short pillar ((NH_2_(CH_2_)_*n*_NH_2_, *n* = 4 and 8) is found to promote the absorption of CO_2_, while a high pillar ((NH_2_(CH_2_)_12_NH_2_) has an unfavorable effect on the CO_2_ absorption. The corresponding micropore surface areas for IGO-*n* (*n* = 4, 8, and 12) are found to be 194.1, 138.4, and 132.5 m^2^ g^−1^, respectively, which are much larger than those determined by N_2_ adsorption. This suggests that some ultramicropores in the interlayer space could be probed by CO_2_ molecules but N_2_ molecules, due to a smaller kinetic diameter of CO_2_ (0.33 nm) than N_2_ (0.36 nm) and a high kinetic energy for CO_2_ diffusion at 273 K. It has been well documented that microporosity plays a key role in CO_2_ adsorption on various carbon materials. The pore size distribution of IGOs was obtained by using the NLDFT method (Fig. 5). Among three IGOs, the IGO-4 has the broadest micropore size distribution in the range of 0.45–0.87 nm, followed by IGO-8 (0.45–0.82 nm), and IGO-12 (0.45–0.78 nm). The cumulative pore volume decrease from 0.036 cm^3^ g^−1^ (IGO-4) to 0.022 cm^3^ g^−1^ (IGO-8) to 0.009 cm^3^ g^−1^ (IGO-12), respectively (Table 1 and Additional file 1: Figure S3). The CO_2_ uptake presents a good correlation with the volume and surface area of ultramicropores. Our observation is also consistent with recent studies on a series of carbon materials that showed enhanced CO_2_ uptake for samples having micropores below 0.7 nm [30–32], where CO_2_ adsorption inside these pores takes place by micropore filling instead of layer by layer adsorption. The declined tendency of micropores for IGO-*n* (*n* = 4, 8, and 12) may be due to the increase of the interplanar space and the decrease of the oxygen-containing functional groups.

After hydrazine reduction, the micropore surface area determined by CO_2_ adsorption is found to be 334.7, 148.1, and 118.5 m^2^ g^−1^ for RIGOA-*n* (*n* = 4, 8, and 12), respectively, and the corresponding CO_2_ uptake capacity reaches as high as 1.97, 0.80, 0.60 mmol g^−1^ at 1 atm and 273 K. For RIGOA-*n* (*n* = 4, 8, and 12), all their CO_2_ uptake capacities are larger than that of RGO (~0.28 mmol g^−1^ at 1 atm and 273 K). When the temperature increases to 298 k, the CO_2_ adsorption capacity decreases for both IGO-*n* (0.58, 0.44, and 0.07 mmol g^−1^ at 1 atm for *n* = 4, 8, and 12, respectively) and RIGO-*n* (1.37, 0.66, and 0.29 mmol g^−1^).

**Fig. 6:**
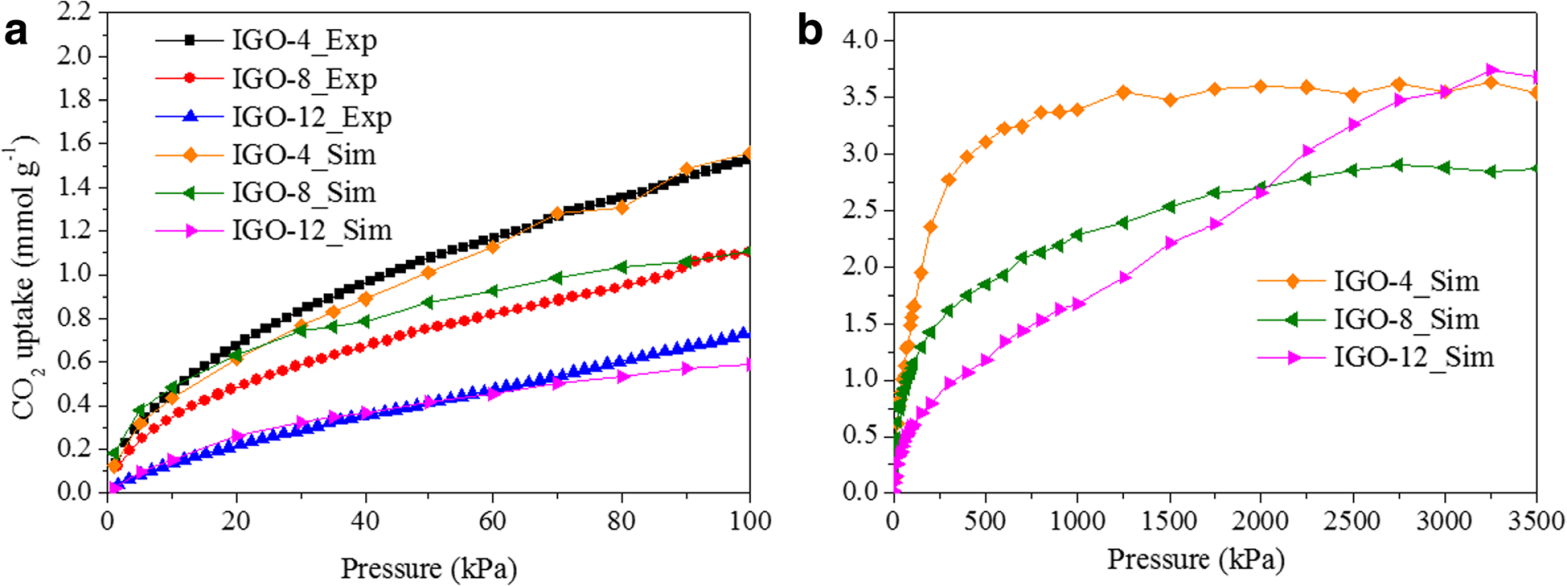
The CO_2_ excess adsorption isotherms for IGOs at 273 K. **a** Both simulated and experimental results at 1–100 kPa. **b** The simulated result at 1–3500 kPa

CO_2_ adsorption in IGOs was also simulated with the Mulliken charges on framework atoms. An atomistic representation of IGOs was built using a DFT computational-assisted structure determination, as described in the “Methods” section. Figure 6 and Additional file 1: Figure S4 present the calculated excess and absolute adsorption isotherms at 273 K using GCMC simulations. It can be seen that the difference between the absolute and excess isotherms increases monotonically at low pressure and tends to flatten out to a constant value of 0.5, 0.8, and 1.0 mmol g^−1^ at 30 bar for IGO-*n*, *n* = 4, 8, and 12, respectively. The largest difference for IGO-12 is attributed to its relatively largest free volume as presence of the maximum interplanar space among three IGOs. At the pressure below 1 atm, the calculated excess adsorption isotherms of CO_2_ are according well with those measured by experiment with a standard deviation of about 0.06 mmol g^−1^, which validates the force fields and models used. The deviation may be attributed to crystal imperfections and presence of solvent molecules inside the pores in synthesized structures. With the increase of pressure, the simulated CO_2_ uptake increases for all IGOs, especially IGO-12. Indeed, the CO_2_ uptake in IGO-12 is even greater than those in both IGO-4 and IGO-8 at 30 bar, and up to 3.68 mmol g^−1^ at 35 bar compared with 3.54 and 2.87 mmol g^−1^ for IGO-4 and 8, respectively. As shown in Fig. 6, CO_2_ uptake of IGO-*n* changes gradually from I- to II-type isotherms for *n* = 4, 8, and 12. Unlike low-pressure uptake which is mostly governed by ultramicropores, large micropores and narrow mesopores are the predominant attributes for attaining high storage capacities at elevated pressures [30, 33]. With the increase of interlayer spacing and decrease of oxygen-containing groups for IGO-*n*, *n* = 4, 8, and 12, the amount of large micropores and narrow mesopores may increase, leading to a larger increase of CO_2_ uptake for IGO-12 than both IGO-4 and IGO-8.

**Fig. 7:**
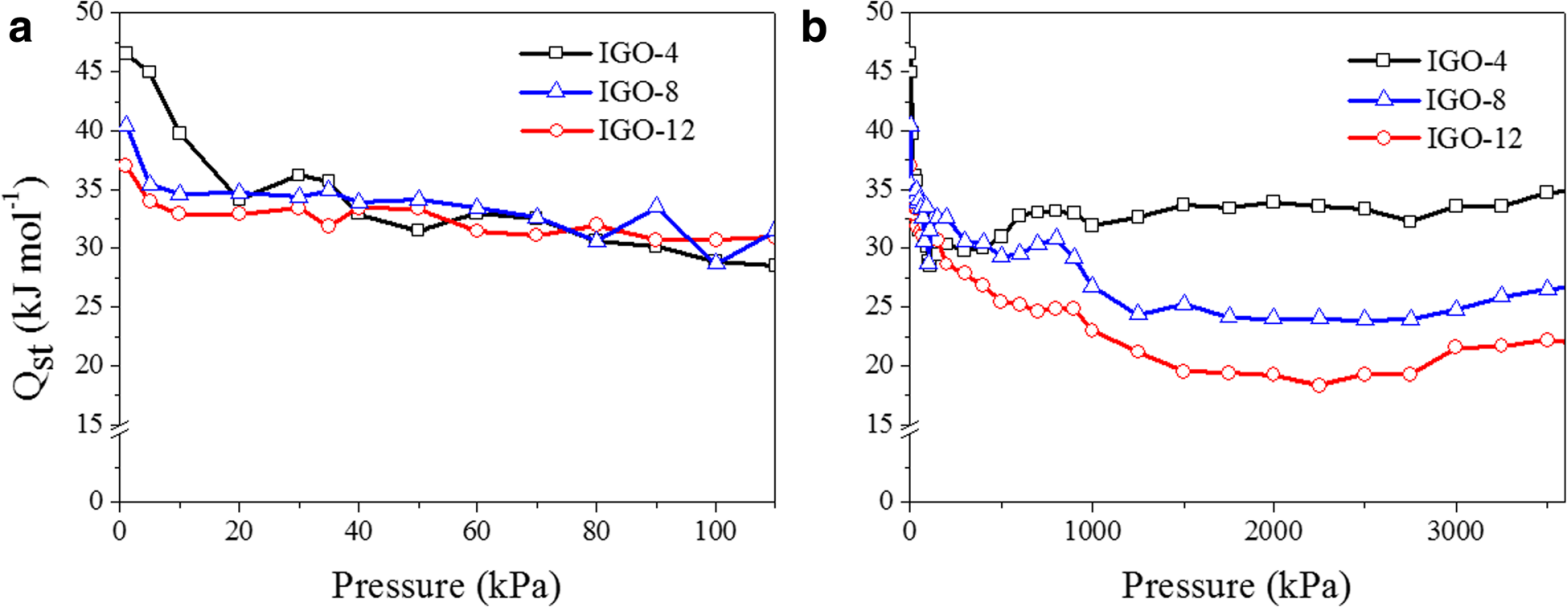
The simulated isosteric heats of CO_2_ adsorption (*Q*_st_) for IGOs at **a** low pressures and **b** high pressures

To investigate the CO_2_/IGO surface interactions, the isosteric heats of adsorption (*Q*_st_) were calculated by the virial method using CO_2_ adsorption isotherms collected at 273 and 298 K (Fig. 7) [34]. The *Q*_st_ value reflects the interaction strength between CO_2_ and IGOs. At a low pressure, the CO_2_ adsorption on IGOs presents high *Q*_st_ (about 37–47 kJ mol^−1^), indicating that the surface of IGOs strongly interacts with CO_2_ molecules, which is attributed to the strong adsorption property of oxygen-containing functional groups on IGO sheets [35]. With increasing sorption pressure, the curves of *Q*_st_ show a drop, indicating a decrease of interaction strength between the IGOs and CO_2_ as intermolecular interaction of adsorbates with the filling of CO_2_. Furthermore, the value of *Q*_st_ follows the order of IGO-4 > IGO-8 > IGO-12, suggesting that CO_2_ molecules interact most strongly with IGO-4, followed by IGO-8, and then IGO-12. This further indicates that a smaller interlayer spacing favors the CO_2_ uptake, due to the stronger superposition of the van der Waals force given by two adjacent walls [32].

**Fig. 8:**
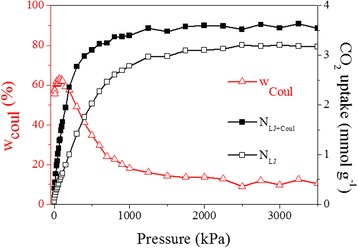
The simulated CO_2_ uptake of IGO-4 at 273 K, with and without the contribution of electrostatic interaction

To further illustrate the effect of electrostatic and van der Waals (non-electrostatic) interactions on CO_2_ uptake, additional simulations were conducted by eliminating the contribution of electrostatic interaction (Fig. 8). As shown in Fig. 8, for IGO-4, the contribution of the electrostatics to the CO_2_ adsorption is relatively large (about 55–65 %) at low pressures and found to be maximum at 0.6 bar. Then, its contribution to adsorption decreases monotonically, contributing a few percent (about 10–14 %) at pressures above 15 bar. Overall, in the presence of electrostatic interaction, the CO_2_ amount adsorbed is about 120–190 % higher than that given by the LJ core alone under low pressures, whereas the adsorption is dominated by the van der Waals force under high pressures.

## Conclusions

We investigated the effect of interlayer spacing on CO_2_ uptake for pillared graphene oxides by both experimental and simulation methods. Interlayer distances of GO were tuned by intercalation of three diaminoalkanes (NH_2_(CH_2_)_*n*_NH_2_, *n* = 4, 8, and 12) with different lengths of alkyl chain. At low pressures, the CO_2_ adsorption capacity of IGOs decreases with the increase of the interlayer distance, where the electrostatic interaction of adsorbent has a larger contribution to the adsorption than van der Waals force. As the pressure increases, CO_2_ uptake of IGO-12 increases sharply and surpasses those of both IGO-4 and IGO-8 at 30 bar, where the van der Waals force plays a dominant role. This new finding demonstrates that the modulation of interlayer spacing of pillared graphene oxides could enhance their CO_2_ adsorbability, which provides useful information to design graphene-based materials with superior CO_2_ adsorption capacity.

## Additional file

Additional file 1: Figure S1. The optimized models of (a) IGO-4, (b) IGO-8, (c) IGO-12. **Figure S2.** The C 1s XPS spectra of GO. **Figure S3.** The cumulative pore volume curves of (a) IGOs and (b) RIGOs determined by CO_2_ sorption. **Figure S4.** The simulated CO_2_ absolute adsorption isotherms for IGOs at 273 K. **Table S1.** Interatomic potential parameters and partial charges for CO_2_ molecule. **Table S2.** Interatomic potential parameters (from the UFF force field) for the atoms in IGOs. **Table S3.** The C 1s XPS spectra of GO.
