# Supplementary material for: The CO2 Storage Capacity of the Intercalated Diaminoalkane Graphene Oxides: A Combination of Experimental and Simulation Studies
Source: Nanoscale Res Lett. 2015 Aug 8;10:318. doi: 10.1186/s11671-015-1026-9 (PMC4529426; doi:10.1186/s11671-015-1026-9)
Supplement: Additional file 1: Figure S1. — The optimized models of (a) IGO-4, (b) IGO-8, (c) IGO-12. Figure S2. The C 1s XPS spectra of GO. Figure S3. The cumulative pore volume curves of (a) IGOs and (b) RIGOs determined by CO2 sorption. Figure S4. The simulated CO2 absolute adsorption isotherms for IGOs at 273 K. Table S1. Interatomic potential parameters and partial charges for CO2 molecule. Table S2. Interatomic potential parameters (from the UFF force field) for the atoms in IGOs. Table S3. The C 1s XPS spectra of GO. [file 11671_2015_1026_MOESM1_ESM.docx]

**Additional file 1**

**The CO_2_ storage capacity of the intercalated diaminoalkane graphene oxides: a combination of experimental and simulation studies**

Jing Xu,^1^ Wei Xing, Lianming Zhao,^1*^ Feifei Guo,^1^ Xiaozhong Wu,^1^ Wenbin Xu,^1^ Zifeng Yan^2*^

*^1^ College of Science, China University of Petroleum, Qingdao, Shandong 266580, PR China*

*^2^ State Key Laboratory of Heavy Oil Processing, Key Laboratory of Catalysis, China University of Petroleum, Qingdao 266580, PR China*

*Correspondence should be addressed to lmzhao@upc.edu.cn (L. Z.);* [*zfyancat@upc.edu.cn*](mailto:zfyancat@upc.edu.cn) *(Z. Y.)*

**Fig. S1** The optimized models of (a) IGO-4, (b) IGO-8, (c) IGO-12.

**Fig. S2** The C 1s XPS spectra of GO.

**Fig. S3** The cumulative pore volume curves of (a) IGOs and (b) RIGOs determined by CO_2_ sorption.

**Fig. S4** The simulated CO_2_ absolute adsorption isotherms for IGOs at 273 K.

**Table S1** Interatomic potential parameters and partial charges for CO_2_ molecule.

**Table S2** Interatomic potential parameters (from the UFF force field) for the atoms in IGOs.

**Table S3** The C 1s XPS spectra of GO.

Fig. S1 The optimized models of (a) IGO-4, (b) IGO-8, (c) IGO-12.

Fig. S2 The C 1s XPS spectra of GO.

Fig. S3 The cumulative pore volume curves of (a) IGOs and (b) RIGOs determined by CO_2_ sorption.

Fig. S4 The simulated CO_2_ absolute adsorption isotherms for IGOs at 273 K.

Table S1 Interatomic potential parameters and partial charges for CO_2_ molecule.

| Atom type | *σ* (Å) | *ε/k_B_* (K) | *q* (e) |
| --- | --- | --- | --- |
| C_CO2 | 2.80 | 27.00 | 0.70 |
| O_CO2 | 3.05 | 79.00 | -0.35 |

Table S2 Interatomic potential parameters (from the UFF force field) for the atoms in IGOs.

| Atom type | C | H | O | N |
| --- | --- | --- | --- | --- |
| *σ* (Å) | 3.43 | 2.57 | 3.12 | 3.26 |
| *ε/k_B_* (K) | 29.13 | 22.12 | 34.72 | 34.75 |

Table S3 The C 1s XPS spectra of GO.

| Bonds | B.E (eV) | Content (%) |
| --- | --- | --- |
| C-C/C=C | 284.6 | 50.9% |
| C-OH | 285 | 7.4% |
| C-O-C | 286.7 | 31.5% |
| C=O | 287.6 | 8.0% |
| O-C=O | 289 | 2.3% |
